# Unraveling the genetic complexity of Alzheimer's disease across diverse populations via integrative single‐cell multi‐omics

**DOI:** 10.1002/alz70855_099090

**Published:** 2025-12-23

**Authors:** Elliot Keats Shwab, Daniel Gingerich, Dellila Hodgson, Zhaohui Man, Melanie E Garrett, Gregory Crawford, Allison Ashley‐Koch, Ornit Chiba‐Falek

**Affiliations:** ^1^ Duke University School of Medicine, Durham, NC, USA

## Abstract

**Background:**

The multifactorial and heterogenous nature of Late Onset Alzheimer's disease (LOAD) presents a challenge, particularly in capturing genetic complexity across diverse populations. Recent single‐nucleus (sn)multi‐omics analyses have advanced the LOAD genetics field. However, most studies were conducted in European ancestry subjects, while other populations remain largely understudied. Here, we aimed to explore the underpinning genetics of LOAD in diverse populations and to gain insights into the shared (pan‐ethnic) and distinct (ancestry‐specific) genetic drivers of LOAD between European and African ancestries.

**Methods:**

We analyzed cortical tissues from European (EA) and African ancestry (AA) LOAD and control donors, and *simultaneously* characterized their transcriptomic (snRNA‐seq) and chromatin accessibility (snATAC‐seq) profiles at a single‐cell level using 10x Genomics *Multiome* technology. We analyzed these datasets using our integrative genomic pipeline to catalogue differentially expressed genes (DEGs) in LOAD and to identify candidate *cis*‐regulatory elements (cCREs) and their target DEGs at the cell‐subtype level. Finally, we performed differential‐expression analysis on the combined snRNA‐seq datasets from both ancestries and modeled ancestry interactions for statistically robust inference of shared and divergent DEGs between ancestries.

**Results:**

EA and AA nuclei were clustered into 32 cell‐subtypes each, representing 8 major neuronal and glial cell types. The highest numbers of DEGs were found in GABAergic and interneuron subtypes in EA vs. GABAergic neuron and oligodendrocyte subtypes in AA. Analysis of cCRE‐DEG pairs revealed differential chromatin interactions governing gene dysregulation across ancestries. For example, microglial *APOE* expression was predicted be regulated by six EA‐specific and four AA‐specific cCREs, and only one common cCRE. Differential expression analysis revealed the highest numbers of pan‐ethnic DEGs in specific excitatory and inhibitory neuronal subtypes, with excitatory‐neuron DEGs primarily associated with cellular growth and extracellular matrix assembly, and inhibitory‐neuron DEGs largely associated with membrane trafficking. Ancestry‐specific DEGs were more commonly identified in AA compared to EA. AA‐specific DEGs were also primarily in excitatory‐neurons, with roles in fatty acid metabolism and neuronal structure, and in inhibitory‐neurons with roles in respiration and synaptic transmission.

**Conclusions:**

These results enhance our understanding of the shared and distinct cell‐subtype gene dysregulation networks and biological processes underlying LOAD in African vs. European ancestries.

